# Diseases of the Intestines

**Published:** 1896-03-21

**Authors:** 


### DISEASES OF THE INTESTINES.

**Anchylostoma Duodenale.**—Some months ago reference was made to a paper by Dr. Sandwith upon the subject of the frequency of anchylostoma duodenale in Egypt. Dr. Sandwith stated that as many as 9·5 per cent. of intending recruits for the army were rejected on account of anæmia produced by this intestinal parasite. Dr. Hayman Thornhill[1] has published an interesting paper drawing attention to the number of natives attacked in Ceylon. He thinks that the percentage of adult males suffering from advanced anæmia due to this parasite is at least 19 per cent., instead of 9·5 per cent. as in Egypt. That being the case, Dr. Thornhill thinks that energetic measures should be taken to prevent infection. Dr. Sandwith was disposed to think that the ova or the larvæ are conveyed in the mouth of an individual by soil-stained hands. Dr. Thornhill thinks also that the ova gain entrance to the body through the soil rather than through the medium of water. Whether, however, conveyed by earth or by water, Dr. Thornhill thinks it matters little, since much the same preventive means should be taken in either case. He recommends the construction of pit latrines in villages and hamlets, and that the natives be induced to use them. He thinks also that power should be given to prevent anyone committing a nuisance within one hundred yards of his own or any other house. As to treatment, Dr. Thornhill prefers male fern to thymol. Not only in his experience is it slightly more efficacious, but it is less disagreeable, and not so dangerous. Four fatal cases of collapse have been recorded during its use, and to these four Dr. Thornhill adds another three.

**Intestinal Antisepsis.**—The question of intestinal antisepsis still seems in a somewhat unsettled state. At a discussion amongst members of the Therapeutical Society of Paris, three of the speakers seemed to think it likely to do more harm than good. Dr. Ferrand,[2] however, who introduced the subject, while he thought that too much had been expected, considered this method of treatment to be moderately successful. Calomel, in his opinion, is most efficacious as an intestinal antiseptic. In order to judge of the value of this or of any other drug, the diminution of toxicity of the urine should be observed. Surveyor and Vaughan Harley[3] have published the results of experiments as to the action of betanaphthol and bismuth subnitrate as intestinal antiseptics, which seem to show that both are of some value, perhaps the former being rather the stronger of the two. It is interesting to find that so old-fashioned a remedy as bismuth can be proved in the laboratory to have considerable power, especially since nowadays there seems to be a tendency in some quarters to look upon it as a drug that has obtained unmerited favour. While, however, bismuth is an intestinal antiseptic, it also has a marked tendency to constipate, which latter action tends in some measure to neutralise the former, by causing delay in the passage of fæces through the alimentary canal. Drs. A. Eilbert and S. A. Dominici[4] show that purgatives, in virtue of what might be called their scavenging action, are of value as antiseptics. They quote the case of a man whose fæces were estimated to contain about twelve millions of microbes per diem. He was given fifteen grammes of sodium sulphate and fifteen grammes of magnesium sulphate, which produced six evacuations of the bowels in the day, containing about 410,000 millions of microbes, or thirty-four times the normal amount. On the second day after the aperients the fæces had resumed their normal external characters, and although large in amount were poor in microbes, containing only one-twentieth of the normal. In connection with the subject of intestinal antisepsis, the experiments of Cassin and Charrin[5] on the protective action of the intestinal mucous membrane are of interest. They mention, in the first place, that toxins introduced into the alimentary canal have a less serious effect upon the animal than when introduced into the general circulation. It has been thought that the passage through the liver renders them more or less harmless. These writers, however, say that the destruction of intestinal toxins does not take place in the liver only, but that the mucous membrane of the intestines themselves has protective power. If the superficial layers of this membrane be destroyed, either by caustics or other forms of mechanical injury, it is found that a certain quantity of toxin introduced into the alimentary canal kills the animal much more quickly.

[1] Indian Medico. Chirurg. Review, Dec., 1895. [2] Med. Week, Dec. 27th, 1895. [3] Lancet, Dec. 14th, 1895. [4] Med. Week, Dec. 27th, 1895. [5] Ibid., Dec. 27th, 1895.

